# Cost-effectiveness analysis of hearing screening program for primary school children in southern Iran, Shiraz

**DOI:** 10.1186/s12887-022-03384-1

**Published:** 2022-05-30

**Authors:** Mohammad Faramarzi, Sara Babakhani fard, Mohsen Bayati, Fatemeh Jafarlou, Mohammadreza Parhizgar, Mehdi Rezaee, Khosro Keshavarz

**Affiliations:** 1 Otolaryngology Research Center, Department of Otorhinolaryngology, School of Medicine, Shiraz University of Medical Sciences, Shiraz, Iran; 2 Student Research Committee, School of Health Management and Information Sciences, Shiraz University of Medical Sciences, Shiraz, Iran; 3 Health Human Resources Research Center, Department of Health Economics, School of Health Management and Information Sciences, Shiraz University of Medical Sciences, Shiraz, Iran; 4 Department of Audiology, School of Rehabilitation Sciences, Tabriz University of Medical Sciences, Tabriz, Iran; 5 Department of Audiology, University of Social Welfare and Rehabilitation Sciences, Tehran, Iran; 6 Department of Health Management, Policy and Economics, Faculty of Management and Medical Information Sciences, Kerman University of Medical Sciences, Kerman, Iran; 7 Emergency Medicine Research Center, Shiraz University of Medical Sciences, Shiraz, Iran

**Keywords:** Screening, Cost-effectiveness, Hearing loss, Primary school

## Abstract

**Background:**

Hearing loss is the second most common chronic disease, the diagnosis and treatment of which can be faster through screening. In addition, early interventions will save significant costs for the education and health systems. Therefore, the present study aimed to evaluate the cost-effectiveness of hearing screening for primary school children in Shiraz.

**Methods:**

This cross-sectional economic evaluation of cost-effectiveness was conducted from the perspective of the health system. The study population comprised all seven-year-old children participating in the screening program in Shiraz. The present study dealt only with direct costs. The expected costs and outcomes, as well as the ICER index were estimated using the decision tree model. The study outcomes included averted disability-adjusted life years (DALY) and true identification of hearing loss cases. The robustness of the results was evaluated using the one-way sensitivity analysis. The TreeAge 2020 and Excel 2016 software were also used to analyze the collected data.

**Results:**

The hearing screening data obtained during 6 years (2015–2020) showed that every year, an average of 22,853 children in Shiraz were examined for hearing, of which 260 were true positive (%1.1). The costs of screening and lack of screening were estimated at $30.32 Purchasing Power Parity (PPP) and $13.75 PPP per child, respectively. The averted DALY due to performing hearing screening was estimated at 7 years for each child. The ICER was positive and equal to $ 0.06 PPP for the identified cases and $ 2.37 PPP per averted DALY. The sensitivity analysis confirmed the robustness of the results.

**Conclusions:**

According to the results, although hearing screening for primary school children had more costs and effectiveness, it was considered cost-effective. Therefore, universal screening with high quality and accuracy is recommended.

## Background

Hearing loss is the second most common chronic physical condition [1]. Hearing loss in childhood refers to severe or mild hearing loss in one or both ears, caused by a disorder anywhere in the hearing system and is considered a severe impairment. Hearing loss can be sensorineural, conductive, or a combination of both [2–5].

The consequences of hearing loss include isolation, decreased social activities, feeling of limitation, and increased depression symptoms [6, 7]. If hearing loss in childhood is not detected early enough, opportunities to improve and achieve a healthy life will be lost [8].

Studies have shown that development of auditory nerves requires early auditory stimulation before the age of two [9]. The onset of hearing loss in children can occur at any age. However, if it is not detected within the first 2 years, it may lead to a communication defect that is difficult to improve with subsequent rehabilitation methods [10]. Various studies have suggested that appropriate detection and intervention for infants with hearing loss should be performed in the first 6 months of life. According to the Joint Committee on Infant Hearing (JCIH) and some other studies, detection at the age of three to 6 months and appropriate interventions at the age of 6 months or earlier could help improve the infant’s life [11–14].

The prevalence of hearing loss in different countries ranges from 0.5 to six per 1000 children [15]. In Iran, however, it is five per 1000 live births [16]. A study in Iran showed that in 2004, the prevalence of bilateral hearing loss was 2.3 per 1000 children [17].

Early diagnosis and treatment of hearing loss in children are the significant benefits of the universal hearing screening program that may enable early interventions in the first years of life to enhance hearing and promote speech development [18, 19]. It can also affect other patient-related parameters such as quality of life and socio-communicative, educational, emotional, and professional development, and may have secondary effects on the children and their families [17, 20]. Hearing screening aims to diagnose hearing disorders shortly after birth to begin the treatment process earlier and allow the suffering child to enjoy normal progress in life [3, 20, 21].

In addition to affecting the quality and development of people’s lives, hearing loss has a significant economic burden. The World Health Organization (WHO) estimated that the global cost of undiagnosed hearing loss was 750–790 billion international dollars (Int $) per year [22]. According to a study, early detection of the disease and the interventions that lead to improved speech can significantly save the costs of the education system and lead to a 10% reduction in the total cost of special education (i.e., more than 50,000 dollars) [23].

There is no doubt about performing Universal Newborn Hearing Screening (UNHS) and it is highly recommended [14, 24–26]. However, the value of other global screening programs is still unclear. The question is whether a screening program is necessary to identify children’s hearing problems when they enter primary school [21] and the School Entry Screening program is a proper intervention for the appropriate use of scarce health resources [27]. Thus, the present study aimed to evaluate the cost-effectiveness of a universal hearing screening program for primary school students in Shiraz. To this aim, the costs, effectiveness, and cost-effectiveness of not screening were compared with those of screening.

## Methods

### Study design and population

This study is a cross-sectional economic evaluation of cost-effectiveness and retrospective research to evaluate the cost and effectiveness of two approaches, including continuity and non-conduct of the hearing screening program for the children entering primary schools in Shiraz. Located in the south of Iran, Shiraz is a metropolis and the capital of Fars province that is approximately between the 27- and 31-degree north orbits and 50- and 55-degree meridians on the east longitude. Having the area of 122,608 km^2^, Fars is the fifth largest province in the country. The capital city of Shiraz with a length of 40 km and a width of 15 to 30 km has an area of 1268 km^2^. It is shaped rectangular and is geographically located in southwestern Iran and in the center of Fars province. The city has a population of about two million and is considered a medical hub in the region. The present study was conducted from the perspective of the health system and no sampling method was used. The research population included all of the children participated in the school entry screening program in 2020 in Shiraz, who were selected through census.

Every year, new students are screened in 13 screening centers in Shiraz. The data of the children screened for hearing within 6 years (2015–2020) was obtained from the Department of Exceptional Education in Shiraz. Furthermore, the information of the children referred to audiologists was used to calculate the prevalence of various types of hearing therapies. To this end, a checklist containing medical information of the children referred at the screening stage was prepared and filled out. Using the results obtained from the checklist, we categorized the children who needed an audiologist or an ENT specialist to receive additional diagnostic tests and treatments.

When enrolling in the first grade of primary school, Iranian children are medically examined in terms of their skin and hair, height and weight, mouth and teeth health, vision and hearing, and academic readiness. One of the services provided is audiometry. School-aged children are screened by the pure tone audiometry method. In this study, all children aged 6 to 7 years who entered the first grade of primary school in Shiraz in 2015–2020 were evaluated. The instruments used in this research were audiometers with frequencies of 500, 1000, 2000, and 4000, as well as tympanometers.

### Model structure

**Fig. 1:**
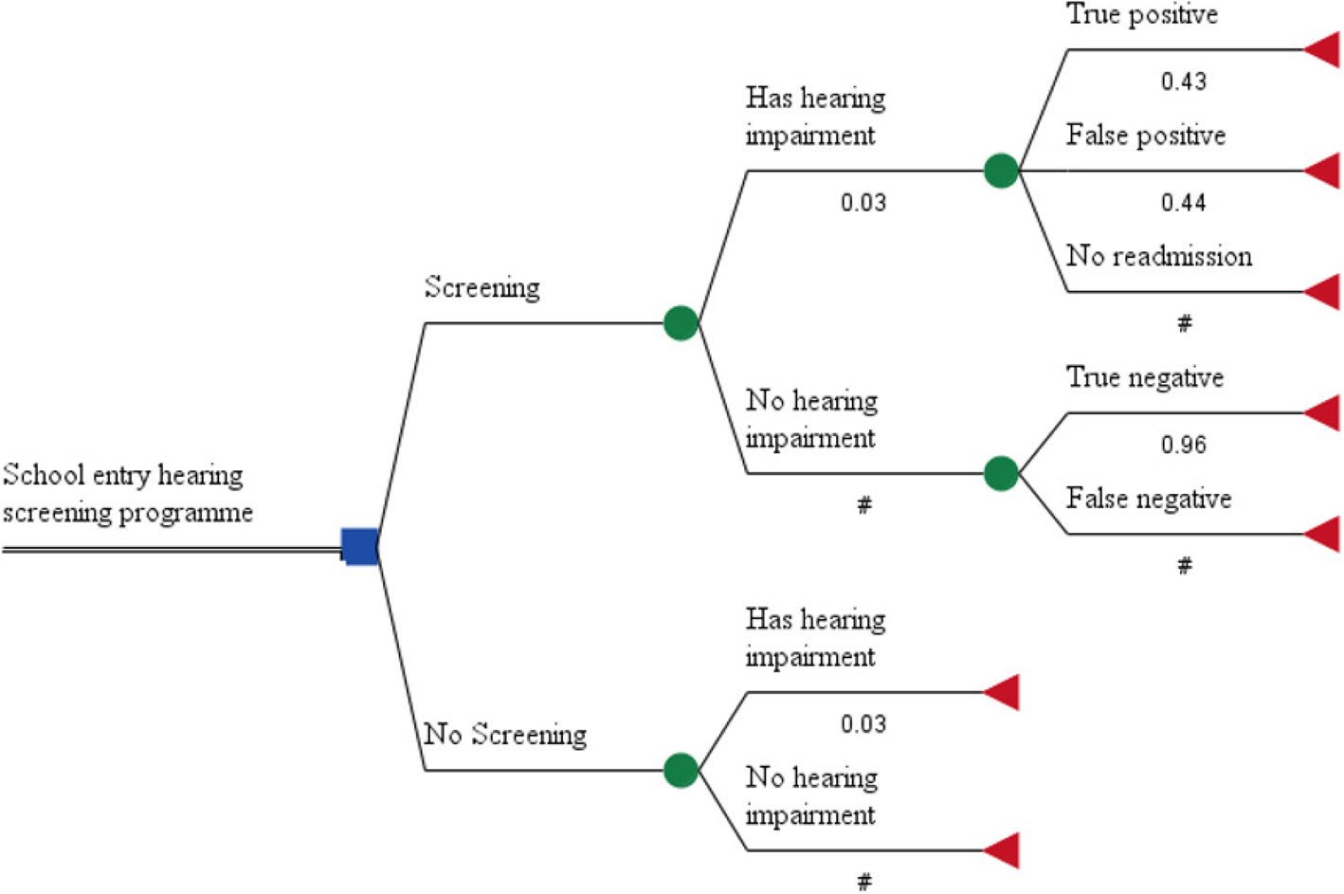
Decision tree for two-steps hearing screening. In this diagram, shows the decision tree model for the two strategies of continuity and non-conduct of the hearing screening program for new primary school children in southern Iran

Figure 1 shows the decision tree model for the two strategies of continuity and non-conduct of the hearing screening program for new primary school children in Shiraz. As presented in the diagram, the costs associated with each strategy were analyzed using the decision tree model and the TreeAge software. For the two screening and no-screening strategies, having and not having hearing problems were considered. When hearing problems were detected through screening, true positive, false positive, and no readmission modes were examined. When no hearing problem was detected, true negative and false negative were examined as well. In case of a true positive, new students were referred to an audiologist if they had a hearing problem. If medical treatment was needed, the audiologist would refer them to an ENT specialist. In case screening was not performed, the family physician was supposed to refer the child to an audiologist or a specialist, if necessary. The related graphs were then analyzed to select the best strategy. In the present research, the audiologists performed screening, evaluation, hearing aid prescription, and final diagnosis, and the patients were referred to an ENT specialist for cochlear implantation and drug therapy if necessary.

### Outcome

The true identification of hearing loss cases and averted DALY were selected as the outcomes for the hearing screening program. It was assumed that the children who were diagnosed with hearing problems after screening and were referred to an audiologist and then to an ENT specialist for further examinations were true cases of hearing loss.

The disability adjusted life years (DALY) were obtained from the sum of years of life lost (YLL) and years of life disabled (YLD). The former was considered zero due to the lack of premature death from hearing loss, and the latter was calculated using the following formula:

$$\mathrm{YLD}=\mathrm{I}\ast \mathrm{DW}\ast \mathrm{L}$$

where I is the number of new cases in the period in question, the disability weight (DW) ranges from zero to one, and L is the mean disability period by year [28].

The DALY was calculated for the conduct of screening and non-conduct. The prevalence of hearing loss and life expectancy was the same in both strategies. The prevalence of hearing loss was 1.14, obtained by dividing the number of true identified cases (260) by the total number of screened cases (22,853) multiplied by 100 (Table 1). According to the WHO, life expectancy in Iran was 74 to 77 years. However, it was estimated at 68.5 years considering the age of the children entering school (7 years old) (https://www.who.int/countries/irn/). The disability weight for the screening strategy was calculated at 0.06 from the lower limit of the geometric mean of different disability levels (mild to severe). In the non-conduct strategy, the disability weight was calculated at 0.15 from the upper limit of the geometric mean of different levels of hearing problems (mild to severe). The upper and lower limits of the disability weight were derived from the study by Orji et al. [29].

**Table 1:** General information on hearing screening in Shiraz during 2015–2020

Variable	All 6 years	Average each year	%
Total number of school-age children who underwent hearing screening	137,115	22,853	100
Number of children referred with hearing loss	3599	600	2.6
Number of children referred for examination after referral	3128	521	2.3
Primary School children with no hearing problems	1364	227	0.1
Primary School children with no hearing problem who had been referred for temporary inflammation of the tympanic membrane, a long interval between the first and the specialized stages, duct collapse, and lack of cooperation, but no hearing problems	205	34	0.1
diagnosed with true hearing problems (true positive)	1559	260	1.1

### Cost

All of the costs were calculated from the perspective of the health system, but only direct costs were estimated. As the decision tree model was used and the study horizon was less than 1 year, the discount rate was not used in this study. The costs of Capital (equipment depreciation) and Current (personnel, consumption, and overhead costs) in the hearing screening program for new students were retrospectively collected for each student from the Department of Exceptional Education of Shiraz in 2020. Other costs, including those of referring the children for hearing tests and treatments, were calculated in consultation with clinical specialists and according to the 2020 tariffs. It was estimated that 70% of the people went to public and 30% to private health centers to receive services. In the present study, short-term costs were calculated based on the screening results, and long-term educational and improvement costs incurred during a person’s life (such as the costs of a special school, liaison teacher, and speech therapy) were not calculated. In addition, the costs were converted to $PPP using the exchange rate of each $PPP equal to 31,317 Rials in 2020 [30].

### Cost-effectiveness analysis

The incremental cost-effectiveness ratio (ICER) was calculated using the ratio of the cost difference to effectiveness difference. The ICER showed how the costs would change with one unit of increase in effectiveness [31]. The following formula was used to calculate ICER:

$$\boldsymbol{ICER}=\frac{\mathbf{Cost}\ \mathbf{A}-\mathbf{Cost}\ \mathbf{B}}{\mathbf{Outcome}\ \mathbf{A}-\mathbf{Outcome}\ \mathbf{B}}$$

The data were analyzed using TreeAge and Excel software.

### Sensitivity analysis

Finally, the one-way sensitivity analysis was used to assess the effects of parameter uncertainty on the results. To perform the one-way sensitivity analysis, some key parameters such as cost and utility were changed by 20% for each strategy, and the results were presented in the form of a Tornado Diagram. Due to the lack of an explicit willingness to pay (WTP) threshold in Iran, and as proposed by the WHO for developing countries, the threshold was one to three times the per capita GDP [32]. The GDP was $ PPP 13,338 [33] in Iran in 2020. Accordingly, the threshold for willingness to pay in the country was $ PPP 40,014 (3* GDP).

## Results

### Outcome

#### 1–1-screening results

**Fig. 2:**
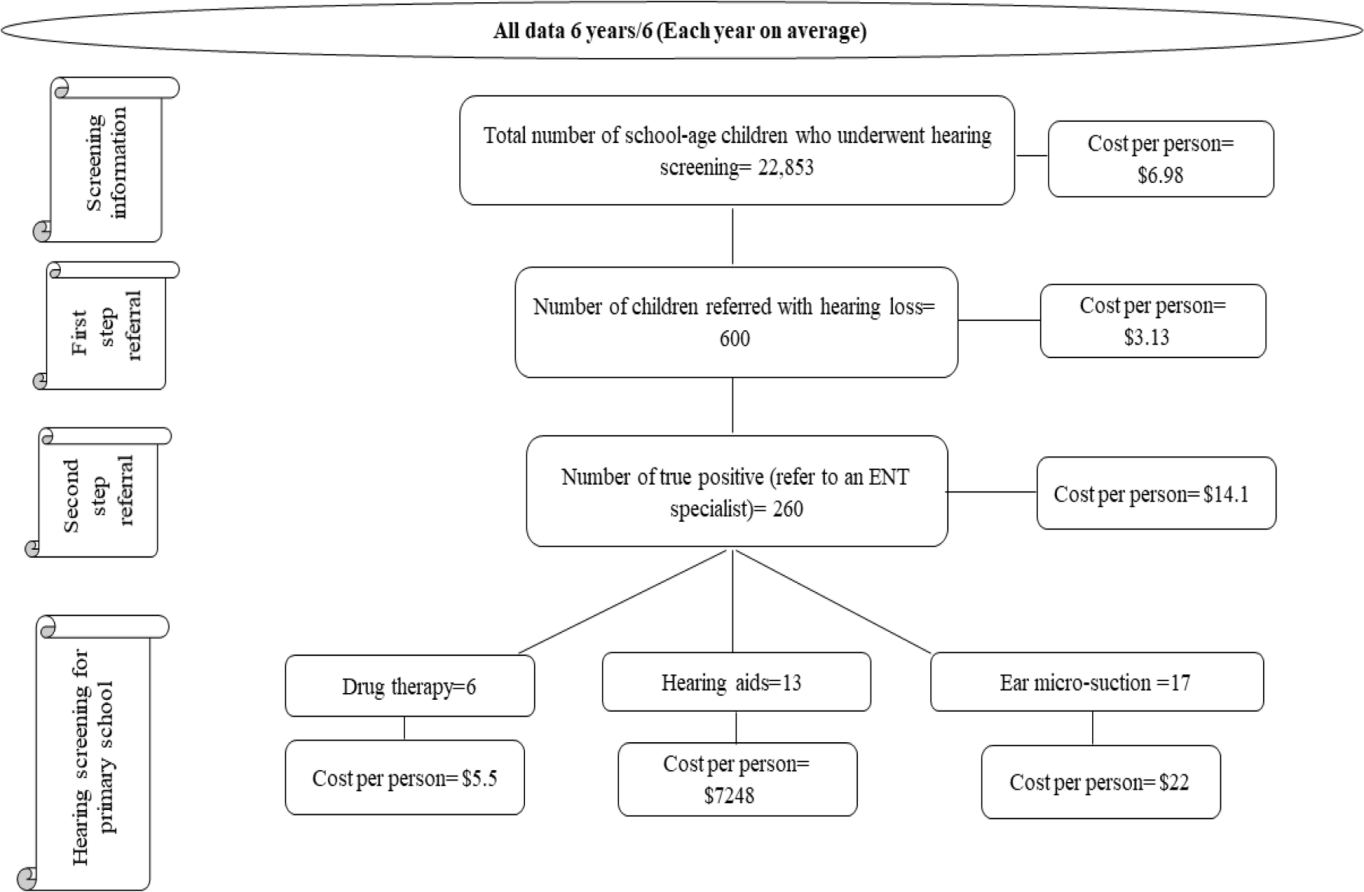
The flowchart of follow-up from screening to problem identification. The diagram shows the mobility of the sample population from the beginning of screening to the time of diagnosis of a hearing problem. * ENT: Ear Nose and Throat Doctor

Figure 2 shows the mobility of the sample population from the beginning of screening to the time of diagnosis of a hearing problem. Table 1 shows general information on young children hearing screening during 2015–2020. The general information of the 6 years is presented in Table 1. The annual data showed that 600 (2.6%) of the 22,853 children examined each year were referred to audiologists, of whom 78.5 (0.3%) did not refer to continue their treatment. Of the 521 (2.3%) children referred, 261 (1.1%) had no hearing problems. However, 260 (1.1%) were diagnosed with true hearing problems (true positive) and were referred to ENT specialists for medical treatment.

Table 2 shows the total number of children diagnosed with true hearing problems (true positive) on hearing screening in Shiraz by type of problem during 2015–2020 (*n* = 1559, average: 260 (1.1%)).

**Table 2:** Total diagnosed with true hearing problems (true positive) on hearing screening in Shiraz during 2015–2020

Variable	All 6 years	Average each year	%
Number of children seeking medical treatment of their hearing problems	453	75.5	29.1
Primary School children referred for ear irrigation	274	45.7	17.6
Primary School children with single-sided hearing loss	132	22.0	8.5
Primary School children who needed hearing aids / in terms of hearing, they could enroll in a regular school	165	27.5	10.6
Primary School children with hearing problems in 1 or 2 frequencies	295	49.2	18.9
Primary School children suffering from hearing loss at a sub-bilateral frequency	25	4.2	1.6
Children who needed hearing aids or had a cochlear implant / had a liaison teacher and were referred to regular schools	113	18.8	7.2
Children either needing hearing aids or having a cochlear implant / going to an exceptional school (also having an intellectual functioning problem)	26	4.3	1.6
Children suffering from sub-single-sided hearing loss	72	12.0	4.6
Children who must enroll in an exceptional school (deaf)	4	0.7	0.3
Total	1559	260	100

Accordingly, the most diagnosed problem is the number of children seeking medical treatment for their hearing problems (*n* = 453, average: 75.5 (29.1%)) and the least diagnosed problems were children who had to enroll in special schools (deaf) due to hearing problems (*n* = 4, average: 0.7 (0.3%)).

Table 3 shows the medical data of the children referred to audiologists in 2020 (*n* = 260). The percentages presented in this table were used to calculate treatment costs.

**Table 3:** Diagnostic information of hearing screening referrals in Shiraz in 2020

Type of Treatment		Number	Percentage
Surgery (all cases were identified before school entry screening)		19	7.3%
Drug therapy	Identification before the age of 6	7	2.7%
	Identification through school entry screening	10	3.8%
Hearing aids	Identification before the age of 6	58	22.3%
	Identification through school entry screening	21	8.1%
Ear micro-suction	Identification before the age of 6	5	1.9%
	Identification through school entry screening	27	10.4%
Healthy		10	3.8%
No need for intervention		82	31.6%
Cochlear implant before 6 years of age		21	8.1%
Total		260	100%

#### 1–2-outcome results

According to Table 2, the number of true cases identified during the 6 years of screening was 1559, which is the sum of the Table 2. On average, there were 260 true positive cases in each year of screening, which was considered as an outcome of the study.

The other outcome of the present study was the averted DALY. Given that the YLL in this study was zero, the DALY was 4.7 in the screening strategy and 11.7 in the non-conduct. Thus, the averted DALY due to screening was 7 years.

### Costs

Table 4 shows the costs of conducting and non-conduct of the hearing screening program per primary school child ($ PPP) from the health system perspective. The results showed that the costs of screening and non-conduct of screening were $30.32 and $13.75 for each new student, respectively.

**Table 4:** Cost information for doing and not doing hearing screening for primary school children in Shiraz in 2020

Costs	Costs items	PPP $
Costs of Hearing Screening Program for primary school children	Capital (Equipment depreciation)	0.51
	Current (Personnel, Consumption and Overhead costs)	4.41
	Total	4.92
Hearing loss diagnostic costs	Visit of the hearing screening audiologist	2.21
	Visit of audiologist	6.43
	Visit of family physician	5.01
	Visit of an ENT specialist	9.94
	Comprehensive audiometric test + TM + AR	16.40
	Total	39.99
Treatment costs for hearing loss	Ear micro-suction	15.51
	Pharmacotherapy	3.88
	hearing aids	5109.84
	Mean cost of ear surgeries	112.10
	Cochlear implant	4694.59
	Total	9935.92

The hearing screening cost per primary school child in 2020 was estimated at $ 4.92.

Diagnostic costs consisted of the costs of visits and tests. The referral fee in the screening program included the cost of visiting an audiologist ($ 2.21 PPP per child) and an audiometric test ($ 16.40 PPP) paid by the government. If screening was not performed, the children were referred to an audiologist ($ 6.43 PPP) by a family physician ($ 5.01 PPP) for required tests ($ 16.40 PPP) and then to a specialist ($ 9.94 PPP) for treatment. Thus, the diagnostic cost per individual would be $ 39.99 PPP. These costs were calculated based on the private and government tariffs set in 2020.

The pharmacotherapy included amoxicillin and co-amoxiclav. The mean cost of drugs was calculated based on the daily consumption and treatment period. Nine common types of auditory practice were identified based on the Relative Values book and the specialists’ opinions. The mean cost of the practices was also calculated.

The treatment costs of hearing screening continuation and non-screening were calculated using the percentages of different treatments obtained through the checklist of those referred to the audiologists in the 2020 screening program (Table 3). In the hearing screening program, hearing problems were recognized to require medication, ear micro-suction, and hearing aids (Table 3). Treatments such as cochlear implants and ear surgery were excluded from hearing screening treatment costs due to the detection and treatment of the problem before entering school.

According to the experts, if primary school children were not screened for hearing problems and were diagnosed late, in the worst case, those who required pharmacotherapy earlier would require hearing surgery in later stages. However, late referral for ear micro-suction and hearing aid treatments would affect the individual’s quality of life and academic achievement (the quality of life was not addressed in this study). Therefore, in the case of non-screening, the costs of ear micro-suction and hearing aids treatments were also considered. Regarding pharmacotherapy, surgical costs (with the percentage of the individuals in need of pharmacotherapy) were considered as well.

The costs of hearing aids, medication, ear micro-suction, and surgery were calculated according to the percentages presented in Table 3 and are shown in Table 5. Thus, the treatment costs of hearing screening and non-screening of school beginners in Shiraz were $ 415.66 PPP and $ 419.67 PPP, respectively.

**Table 5:** Information on medical costs in case of continuation and non-performance of hearing screening program for school beginners in Shiraz city

Costs	Type of treatment	Treatment tariff	Percentage identified through screening	PPP $
Treatment cost of screening continuation	Hearing aids	5109.84	0.081	413.88
	Ear micro-suction	15.51	0.106	1.64
	pharmacotherapy	3.88	0.037	0.14
	Total			415.66
Treatment cost in case of non-screening	Hearing aids	5109.84	0.081	413.88
	Ear micro-suction	15.51	0.106	1.64
	Surgery	112.10	0.037	4.15
	Total			419.67

### Cost-effectiveness analysis

**Fig. 3:**
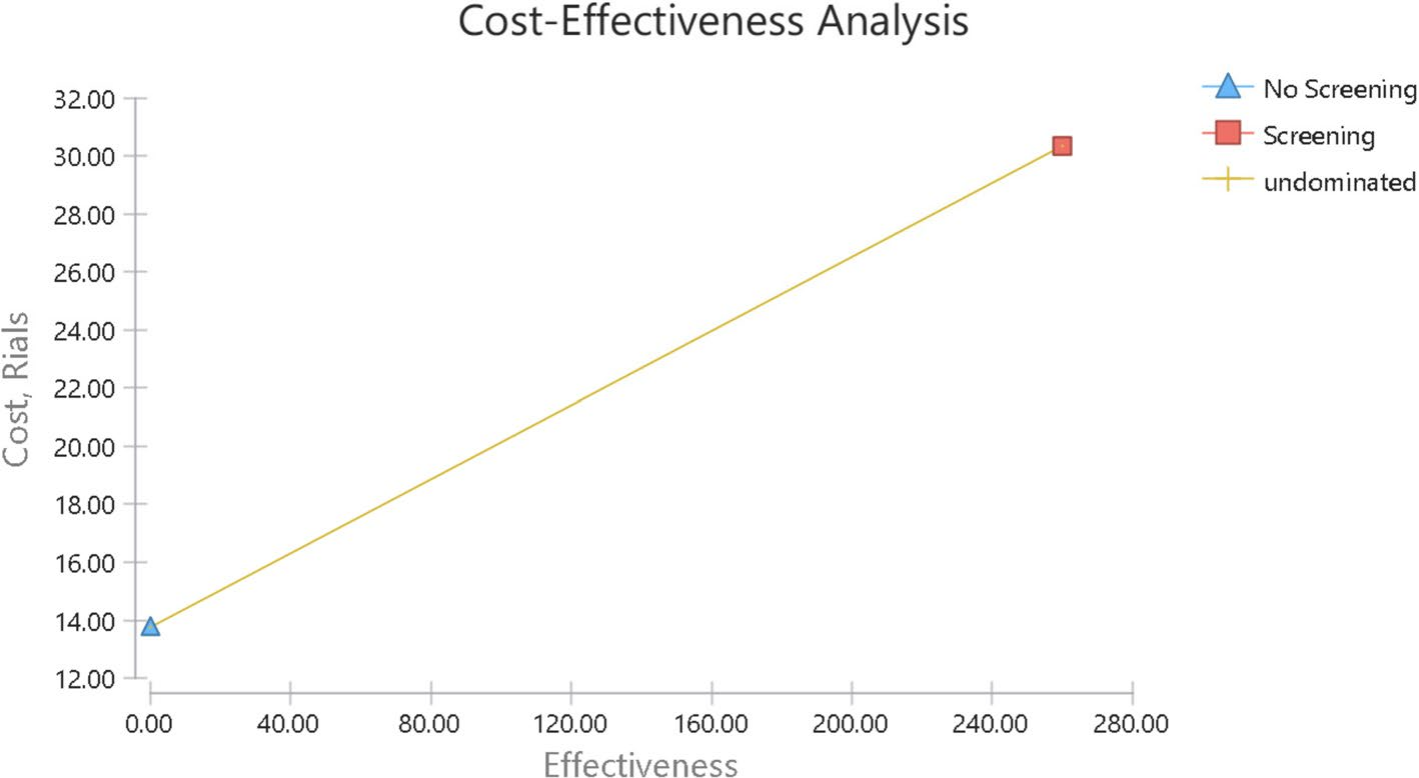
Cost-Effectiveness analysis to the true identification of hearing loss cases of the hearing Screening and no hearing screening children in primary schools. This figure showed that the screening strategy was the superior strategy and more cost-effective than the no screening strategy because the ICER was below the cost-effective threshold

**Fig. 4:**
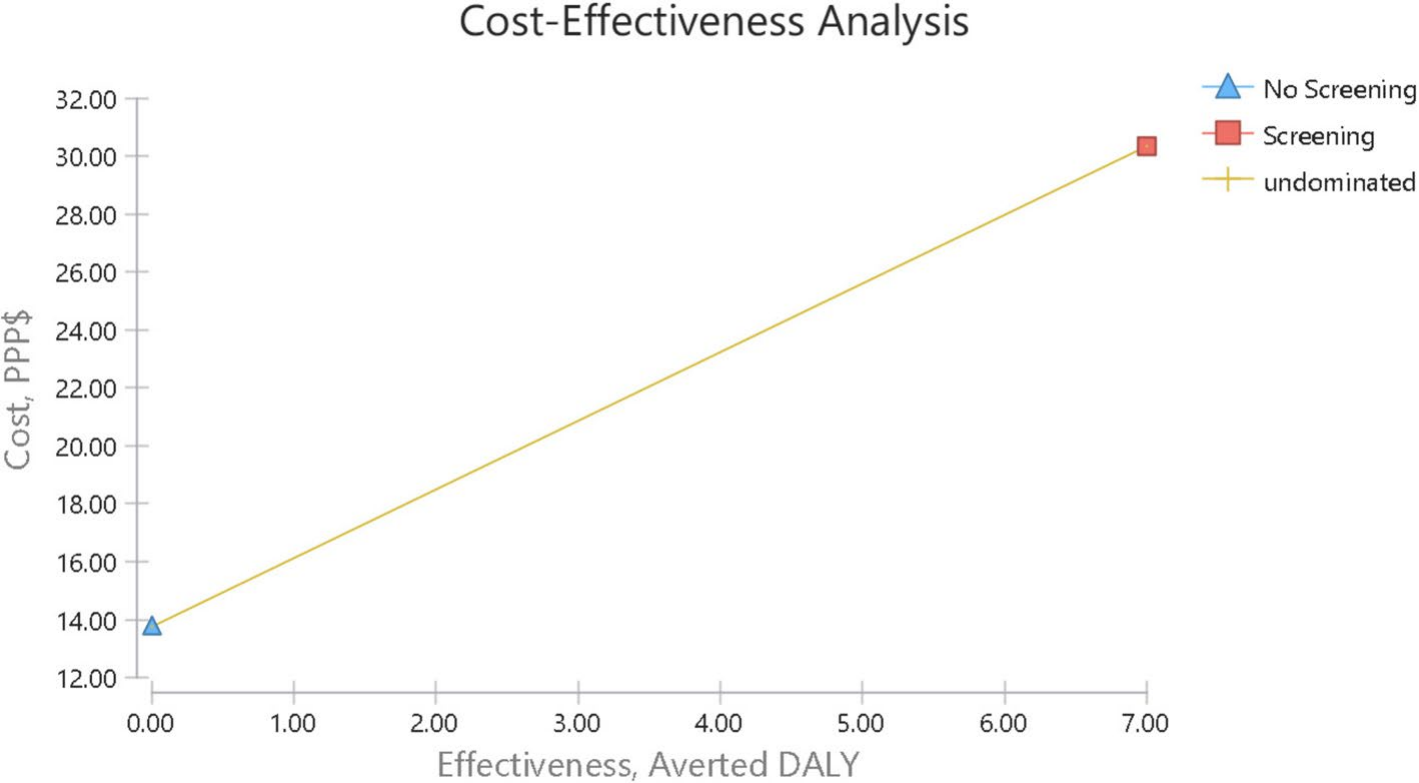
Cost- Effectiveness analysis to averted DALY of the hearing Screening and no hearing screening children in primary schools. This figure showed that the screening strategy was the superior strategy and more cost-effective than the no screening strategy because the ICER was below the cost-effective threshold

The results of the cost-effectiveness analysis (Figs. 3 and 4) showed that although the screening strategy was more costly and more effective, it was the superior strategy and more cost-effective than the no-screening strategy because the ICER in the present study was below the cost-effective threshold.

The cost-effectiveness of the screening and no-screening strategies are shown in Table 6. The former was more effective in terms of the identified cases (*n* = 260) and averted DALY(=7), but the latter had an effectiveness of zero in terms of both items. On the other hand, the cost of the screening strategy ($ 30.32 PPP) was higher than that of no-screening ($ 13.75 PPP). The ICERs calculated for the number of true positive cases and the DALY were $ 0.06 PPP and $ 2.37 PPP, respectively, indicating that the screening strategy was superior despite its positive ICER and higher cost and effectiveness, because its ICER was below the threshold.

**Table 6:** The result of cost-effectiveness in Screening versus No Screening

Strategy	Cost (PPP$)	Effectiveness	averted DALY	Incremental cost	Incremental effectiveness		ICER (PPP$)		Subset
					Effectiveness	averted DALY	Effectiveness	averted DALY	
No screening	13.75	0	0	0	0	0	0	0	dominated
Screening	30.32	260	7	16.57	260	7	0.06	2.37	dominant

### Sensitivity analysis

**Fig. 5:**
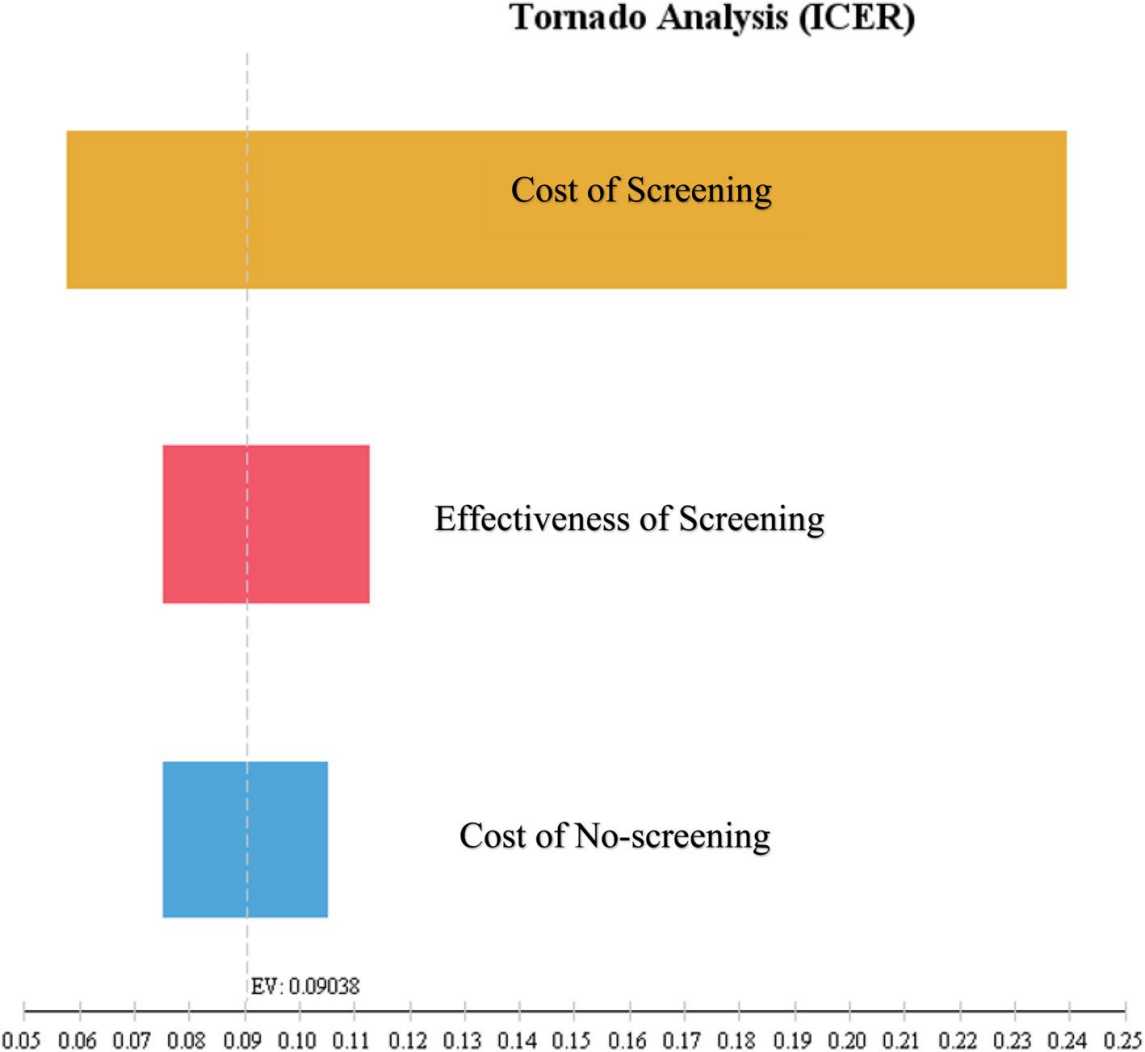
Tornado diagram of cost-effectiveness for screening and no screening. The diagram showed the results of one-way sensitivity analysis. The value of each parameter was changed by 20% and the results are shown by the Tornado diagram. The ICER had the highest sensitivities to the cost of screening

**Fig. 6:**
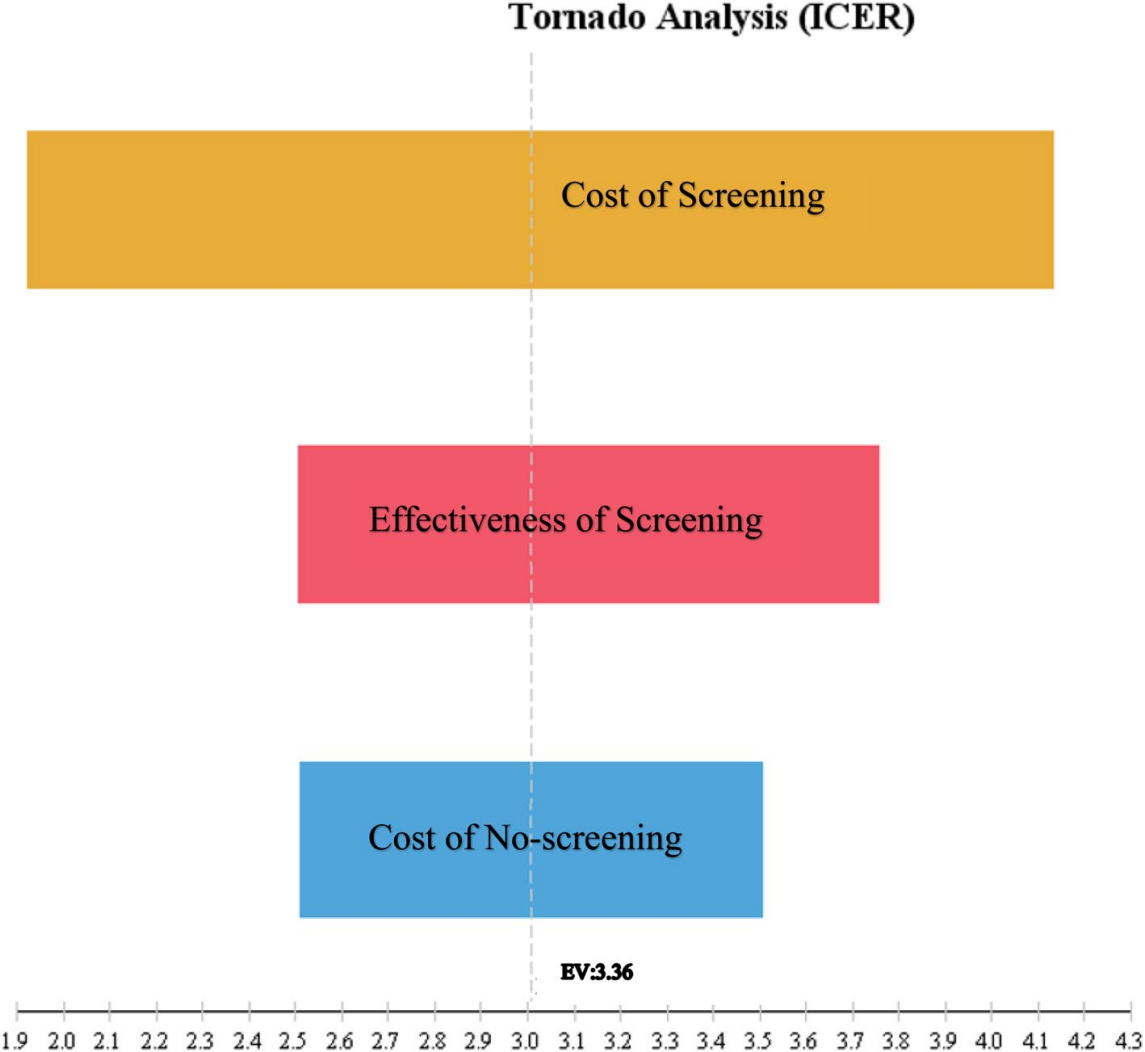
Tornado diagram of cost-utility for screening and no screening. The diagram showed the results of one-way sensitivity analysis. The value of each parameter was changed by 20% and the results are shown by the Tornado diagram. The ICER had the highest sensitivities to the cost of screening

In the one-way sensitivity analysis, the value of each parameter was changed by 20% and a tornado diagram was presented to identify the individual parameters with the highest and lowest sensitivities on the outcome (ICER). The tornado diagram showed that the findings of this study were more sensitive to the cost of screening and had the lowest sensitivity to the cost of no screening (Figs. 5, 6).

## Discussion

As stated, early diagnosis and treatment of hearing disorders to improve academic achievements and social relations are recommended in many studies. The present study aimed to compare the costs and effectiveness of the two strategies, including conducting hearing screening for primary school children and no screening. During 6 years, 22,853 primary school children were screened and on average, 260 true positive cases were identified each year. The prevalence of hearing loss in this study was 1.14, which is consistent with the WHO’s estimates of global disease burden [34]. The study outcomes were the number of true cases identified and the averted DALY, which were 260 and 7, respectively, if the screening program continued, and zero if the children were not screened. As the results indicated, medical expenses accounted for the highest costs ($ 9935.92 PPP). Regarding the deduction of cochlear implant cost and the use of prevalence percentages to calculate medical expenses, the costs were estimated at $ 415.66 PPP and $ 419.67 PPP in the screening and no-screening strategies. However, medical costs were still the highest. The screening costs were the lowest due to the breakdown of the total cost of screening on the types of provided services (checking skin and hair, height and weight, mouth and teeth health, academic readiness, vision, and hearing).

The results of a systematic review of previous research on the cost-effectiveness of preschool hearing screening showed that the costs presented in different studies varied significantly [35]. Subsequently, the results of various studies were reviewed. The present research showed that from the health system perspective, the cost of hearing screening was $ 30.32 PPP and the no-screening cost was $ 13.75 PPP per primary school child. A study by Yin et al. showed that transient evoked otoacoustic emissions (TEOAEs) screening for at-risk preschoolers cost $ 18.03 [36]. One of the main reasons for the difference between the costs in this study and Yin et al.’s is that the present study addressed universal screening, but Yin examined targeted screening. Various studies suggested that general screening was costly. Baltussen and Smith estimated the costs of hearing screening for children and adults without health utilities every 6 years. The results of the passive screening in Southeast Asia showed that the DALY, costs, and ICER were 1207 per million, $1.04 Int, and $ 892 Int [37], respectively. Aasham et al. in Oman estimated the cost of hearing screening for each primary school child at $ 5 and the total cost for each referred child at $ 4700. Given the low prevalence of hearing disorders, they concluded that hearing screening for primary school children was not cost-effective [38]. The screening cost in their study was $ 4.92 PPP and the cost for each referred (true positive) child was $ 635 PPP. Nguyen et al. estimated the cost of screening at $ 88 per child [39].

Fortnum et al. indicated that school entry screening (SES) was unlikely to be cost-effective unless referrals were made faster by the SES [21]. The same result was obtained in other studies [40, 41]. In contrast, reviewing the articles on the cost-effectiveness of school entry hearing screening suggested that most studies showed the cost-effectiveness of the screening strategies [35]. The present study also showed that school entry hearing screening was cost-effective. However, not conducting the screening was not yet discarded, and it should be noted that universal screening was not the best method for hearing screening. Another study indicated that high-error screening could lead to 50% inaccurate identification of hearing loss cases [42]. In addition, according to Fig. 1, 44% of the cases referred in the screening program were false positive. Therefore, the accuracy and quality of screening for primary school students must be taken into consideration.

Other researchers such as Diener et al. (2017) stated that targeted cytomegalovirus (CMV) screening improved early detection of hearing loss from 56 to 77%. Therefore, follow-up and evaluation of risk cases could result in more improvement. Thus, they recommended examining the children with risk factors [43]. CMV is the most common congenital infection and prevalent non-genetic cause of congenital sensorineural hearing loss. Gantt et al. (2016) conducted a similar study and concluded that targeted screening of the children with risk factors was more cost-effective than screening all children [8]. Therefore, children with at least one identifiable risk factor, such as a family history of permanent hearing loss, congenital infections (e.g. cytomegalovirus (CMV), rubella, syphilis), staying in a pediatric intensive care unit or a care unit for infants, neurological disorders or brain development [2], and weighing less than 1500 g at birth [44] could be candidates for the examination. Newton et al. showed the effectiveness of a questionnaire for identifying the children with hearing loss. Using 757 questionnaires completed by the parents, they detected 13 children with bilateral hearing loss whose problem had not previously been diagnosed [45].

The present study did not deal with the impact of hearing loss on individuals’ quality of life. Other studies showed that hearing loss had considerable economic costs, and according to the Centers for Disease Control, the lifetime medical, educational, and occupational costs of hearing loss for the children born in 2000 were $ 2.1 billion [46].

The results of the one-way sensitivity analysis suggested that despite the positive ICER and higher cost and effectiveness, the screening strategy was cost-effective since its ICER was below the threshold. In addition, the highest sensitivity was to the cost of screening.

One limitation of this study was the lack of considering long-term educational and training costs caused by hearing loss and not addressing indirect costs when analyzing the results. Another limitation was not dealing with the inevitable effects of hearing loss on quality of life, academic achievement, career advancement, and social relations.

## Conclusions

The results of the present research and the review of similar studies indicated the importance of the identification of children with hearing disorders before the age of two. Despite the cost-effectiveness of the hearing screening program for primary school children, late detection of hearing disorders when entering school will still prevent them from normal communication and academic progress. Hearing improvement interventions after the age of two are less valuable and effective than early interventions in infancy. Therefore, it is suggested to explore and develop methods for faster detection of hearing loss before the age of two to inform parents. Future studies are suggested to examine and compare the effectiveness of hearing interventions for children at different ages.

## Data Availability

Datasets analyzed during the current study are available from the corresponding author on reasonable request.

## Abbreviations

DALY: Disability adjusted life years
ICER: Incremental Cost-Effectiveness Ratio
WHO: World Health Organization
JCIH: Joint Committee on Infant Hearing
UNHS: Universal Newborn Hearing Screening
PPP: Purchasing Power Parity
YLL: Years of Life Lost
YLD: Years of Life Disabled
DW: Disability Weight
WTP: Willingness To Pay
CMV: Cytomegalovirus
